# The effects of 5-aminolevulinic acid photodynamic therapy on the local immune response of women with cervical intraepithelial neoplasia grade 2

**DOI:** 10.3389/fimmu.2023.1211114

**Published:** 2023-10-20

**Authors:** Anyue Wu, Jing Niu, Zubei Hong, Liying Gu, Yuli Huang, Lihua Qiu

**Affiliations:** ^1^Department of Obstetrics and Gynecology, Renji Hospital, School of Medicine, Shanghai Jiaotong University, Shanghai, China; ^2^Shanghai Key Laboratory of Gynecologic Oncology, Renji Hospital, School of Medicine, Shanghai Jiaotong University, Shanghai, China; ^3^Department of Obstetrics and Gynecology, Chongming Hospital, Shanghai University of Medicine and Health Sciences, Shanghai, China; ^4^State Key Laboratory of Oncogenes and Related Genes, Shanghai Cancer Institute, Renji Hospital, School of Medicine, Shanghai Jiaotong University, Shanghai, China

**Keywords:** photodynamic therapy, CIN2, local immune response, hrHPV, CD8^+^ T cells

## Abstract

**Objective:**

To evaluate and elucidate the effects and mechanism of 5-aminolevulinic acid photodynamic therapy (ALA-PDT) on the local immune response of women with cervical intraepithelial neoplasia grade 2 (CIN2).

**Materials and methods:**

Immunofluorescence staining was used to compare immune cells infiltration before and after ALA-PDT in 23 patients with CIN2. The infiltration of immune cells into the cervical tissues of patients with different outcomes was also compared at the 6-month follow-up period. Immune cell counts in samples collected before and after treatment were compared.

**Results:**

We found an increased number of CD8^+^ T cell infiltration, an increased proportion of CD8^+^ T cells expressing Granzyme B (GrB), Chemokine receptor 3 (CXCR3), and CD8^+^ tissue-resident memory T (T_RM_) cells, and a decreased proportion of CD8^+^ T cells expressing PD-1 in patients with CIN2 compared to that before ALA-PDT. Moreover, at the 6-month follow-up, there was higher infiltration of CD8^+^ T and CD8^+^ T_RM_ cells, higher expression of GrB and CXCR3, and lower expression of PD-1 on CD8^+^ T cells in the HPV clearance and CIN2 disappearance groups than in the HPV-positive and CIN2 regression groups. However, no significant difference was observed in the number of CD8^+^ T_SCM_ following ALA-PDT.

**Conclusion:**

ALA-PDT could activate CD8^+^ T cell responses by modulating the expression of CXCR3 and PD-1 in CD8^+^ T cells and increasing the infiltration of CD8^+^ T_RM_ cells. And the infiltration of CD8^+^ T cells is correlated with the prognosis of CIN2.

## 1 Introduction

Despite being largely preventable through early vaccination and screening strategies, the incidence of cervical cancer ranks fourth among female malignancy (1). It is universally known that high-risk HPV (hrHPV) infection is a major factor that leads to high-grade cervical intraepithelial neoplasia (CIN). CIN2, an intermediate stage between CIN1 and CIN3, is a premalignant state; if left untreated and uncontrolled, it may develop into cervical cancer (2). Therefore, there is an ongoing medical effort to slow the progression of precancerous cervical lesions and reduce the incidence of cervical cancer.

Typical treatments for CIN2 include invasive procedures, such as laser surgery, cryotherapy, cold knife conization, and large loop excision of the transformation zone. Despite their positive effects on CIN2 regression or elimination, several severe side effects are apparent during routine application (3). Thus, a noninvasive therapeutic intervention that eliminates lesion and decreases the progression of CIN2 is desirable. 5-Aminolevulinic acid photodynamic therapy (ALA-PDT) is a non-invasive treatment that has been successfully used to treat tumors and many other diseases, with the advantages of low toxicity and high selectivity. Our previous studies has demonstrated that ALA-PDT is effective on treating hrHPV infection in patients with no cervical lesions (4), and ALA-PDT is a highly effective therapeutic procedure for cervical LSIL with hrHPV infection (5). Furthermore, our previous data showed that ALA-PDT emerge as a promising alternative to observation and surgical procedures in patients with CIN2 (6). Numerous studies have demonstrated that the cytotoxic mechanism of ALA-PDT relies on the accumulation of photosensitizers in the target cells, which generate reactive oxygen species (ROS) to kill abnormal cells (7). Recent studies have shown that ALA-PDT can activate an immune response, which plays a crucial role in clearing lesions. In addition, the immune response induced by ALA-PDT is correlated with therapeutic outcomes (8). Mechanistic studies have revealed that antigens released from dead cells induced by ALA-PDT can be presented by dendritic cells (DCs), which then activate specific CD8^+^ T cell responses (9). There were fewer CD8^+^ T cell infiltration in hrHPV infected and precancerous epithelium compared to normal epithelium, thus the CD8^+^ T cell response helps determine which cervical hrHPV infections persist and progress to precancer and cancer (10, 11). Currently, most studies have focused mainly on immune responses in the tumor microenvironment and splenic lymphocytes, and there are few studies on the effect of ALA-PDT on the local immune response, especially on CD8^+^ T cell response against CIN2.

In this study, we aimed to investigate the effect of ALA-PDT on the local immune response against CIN2 and uncover the underlying molecular mechanisms.

## 2 Methods

### 2.1 Study design

We selected 23 patients with CIN2 who received ALA-PDT at the Renji Hospital of Shanghai Jiaotong University based on the following inclusion criteria: (i) women between the ages of 18 and 50 years; (ii) histologically proven CIN2 before treatment; (iii) has completed ALA-PDT as previously administered (6); (iv) follow-up information at 6 months after treatment; and (v) having been infected with hrHPV. The exclusion criteria were as follows: (i) receiving other treatments such as laser therapy during ALA-PDT and (ii) having immune system and autoimmune diseases.

This study was approved by the Ethics Committee of Renji Hospital (CTR20210524) and was performed in accordance with the Declaration of Helsinki and national and international guidelines. Written informed consent was obtained from all the patients, and all patients were followed up for 6 months.

### 2.2 Data and sample collection

We obtained 23 pairs of archival formalin-fixed paraffin-embedded (FFPE) blocks at pre-treatment and at the 6-month follow-up period. We divided the outcomes of ALA-PDT for patients with CIN2 into four groups at the 6-month follow-up period: disappearance, regression, persistence, and progression of CIN2, and two groups for HPV: HPV clearance and HPV-positive.

The disappearance of CIN2 was defined as the disappearance of initial findings and a normal histology. The regression of CIN2 was defined as the reduction in the severity of the histological results (LSIL or condyloma-like) compared to the initial state histology. The persistence of CIN2 was defined as no change by colposcopy-directed biopsy. The progression of CIN2 was defined as the diagnosis of CIN3+ resulting from a biopsy guided by colposcopy. Moreover, HPV clearance was defined as patients with HPV-negative results and HPV-positive was defined as patients with hrHPV infection at 6 months follow-up.

### 2.3 Multiple immunofluorescence

FFPE tissue blocks were cut into 4 um slides and blank slides were deparaffinized in xylene and rehydrated with an ethanol series. Next, they were incubated with primary antibodies against CD45 (1:50, Santa Cruz sc-28369), CD3 (1:500, Abcam ab237707), CD8 (1:100, Cell Signaling Technology 70306), Granzyme B (GrB) (1:50, Cell Signaling Technology 46890), Chemokine receptor 3 (CXCR3) (1:500, Abcam ab288437), TCF1 (1:50, Cell Signaling Technology 2203), CD45RO (1:400, Cell Signaling Technology 55618), CD103 (1:50, Cell Signaling Technology 95835), and programmed cell death protein 1 (PD-1) (1:100, Cell Signaling Technology 86163) at 4°C overnight. The samples were then incubated with a fluorescence-coupled secondary antibody. Images were captured using a confocal laser scanning microscope (Leica TCS SP8 STED; Leica Microsystems GmbH, Wetzlar, Germany). The fluorescence integral density was measured using ImageJ software (v1.48; National Institutes of Health, USA).

### 2.4 Cell counts

Images were captured using a Pannoramic 250 digital slide scanner (3DHISTECH, Ltd., Budapest, Hungary). Ten areas representing the density of immune infiltrates in each sample were selected. Images of these ten areas were captured on each stained slide using a panoramic viewer at 40×. Cells were manually counted by two individual researchers using the cell counter plug-in in ImageJ software.

The mean cell count per image was then calculated. Cell counts were converted and presented as the number of cells per mm^2^. Each case was assigned a random subject number to ensure that both researchers were unaware of the clinical characteristics of each case when analyzing the immune infiltrate.

The infiltration of CD8^+^ T cells in CIN2 was compared before and after ALA-PDT in patients with different outcomes at the 6-month follow-up. We used the median cell counts of CD45, CD3, CD8, GrB, CXCR3, CD103, CD45RO, TCF1, and PD-1 in patients with CIN2 (n = 23).

### 2.5 Statistical analysis

All results are expressed as mean ± standard deviation (SD). An unpaired Student’s t-test was performed for comparisons between two groups. All analyses were performed using the GraphPad Prism 7 software (GraphPad, San Diego, CA, USA). Statistically significant differences between groups were defined as *p* < 0.05.

## 3 Results

### 3.1 Patient characteristics

We selected 23 patients with CIN2 who underwent ALA-PDT. The mean age was 27.65 ± 4.35 years old (20–48 years old). At the 6-month follow-up, 16 of the 23 patients treated with ALA-PDT achieved complete hrHPV clearance. In addition, the CIN2 disappeared in 15 patients, regressed in 8, and persisted or progressed in 0. See **Table 1** for an overview of patient characteristics.

**Table 1:** Baseline patient: clinical characteristics and patient outcome.

	Pre-PDT treatment			Post-PDT treatment		
Patient	HPV	Cytology(TCT)	Histology	HPV	Cytology(TCT)	Histology
1	18 52 56	NILM	CIN II	18 52	NILM	Condyloma-like
2	18 51 53	ASCUS	CIN II	neg	NILM	No dysplasia
3	52	NILM	CIN II	neg	NILM	No dysplasia
4	68	NILM	CIN II	neg	NILM	No dysplasia
5	31	ASCUS	CIN II	31	NILM	Condyloma-like
6	51 52 39	NILM	CIN II	neg	NILM	No dysplasia
7	35	NILM	CIN II	neg	NILM	No dysplasia
8	16	NILM	CIN II	neg	NILM	No dysplasia
9	58	NILM	CIN II	neg	NILM	No dysplasia
10	16 44	NILM	CIN II	neg	NILM	No dysplasia
11	33 52 44	LSIL	CIN II	33	NILM	No dysplasia
12	56	NILM	CIN II	neg	NILM	No dysplasia
13	16	NILM	CIN II	neg	NILM	Condyloma-like
14	16	NILM	CIN II	39	NILM	No dysplasia
15	52	NILM	CIN II	neg	NILM	No dysplasia
16	16	ASCUS	CIN II	neg	NILM	No dysplasia
17	16 6	ASCUS	CIN II	68	NILM	Condyloma-like
18	16	ASC-H	CIN II	16	NILM	LSIL
19	16	NILM	CIN II	52	NILM	No dysplasia
20	33	NILM	CIN II	neg	NILM	No dysplasia
21	33 6	NILM	CIN II	neg	NILM	LSIL
22	16	NILM	CIN II	neg	NILM	Condyloma-like
23	58	LSIL	CIN II	neg	NILM	Condyloma-like

### 3.2 ALA-PDT increases infiltration of CD8^+^ T cells

**Figure 1:**
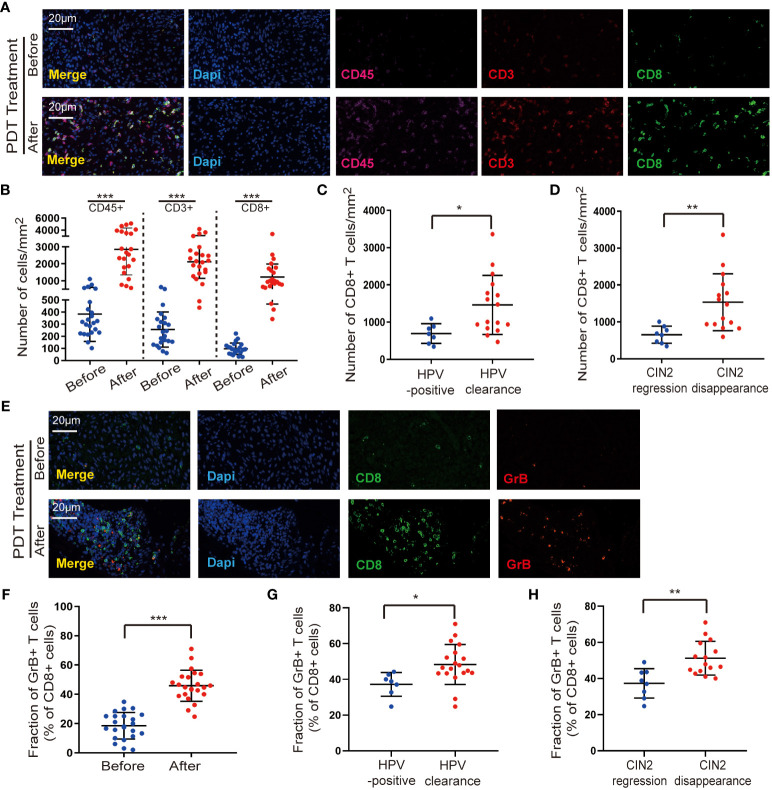
ALA-PDT increases the infiltration of immune cells. **(A, B)** Lesions contained significantly higher numbers of CD45^+^ leukocytes and CD8^+^ T cell infiltration after ALA-PDT treatment (*p* < 0.001). **(C)** At the 6-month follow-up, the number of CD8^+^ T cell subsets was significantly higher in the HPV clearance group than in the HPV-positive group. **(D)** At the 6-month follow-up, the number of CD8^+^ T cell was higher in the CIN2 disappearance group than in the CIN2 regression group. **(E, F)** An increase in the percentage of CD8^+^ T cells expressing GrB was observed in the post-treatment samples. **(G)** The proportion of CD8^+^ T cells expressing GrB was significantly higher in the HPV clearance group than in the HPV-positive group. **(H)** The CIN2 disappearance group showed higher GrB expression of CD8^+^ T cells than the CIN2 regression group. Differences between linked groups were evaluated by two-tailed Student’s t test. ^*^p<0.05; ^**^p<0.01; ^***^p < 0.001.

To analyze changes in the variety and quantity of immune cells after ALA-PDT, we performed immunofluorescence staining. CD45 is a receptor-linked protein tyrosine phosphatase that is expressed in all leukocytes; thus, we first examined the expression of CD45 in tissues. We observed a small amount of CD45^+^ immune cell infiltration in untreated CIN2, and lesions contained significantly (*p* < 0.001) higher numbers of CD45^+^ leukocytes after ALA-PDT (**Figures 1A, B**). According to a previous study (12), a better understanding of HPV tumor–host immune system interactions is mainly related to T cell-based immunity directed toward the elimination of viral-infected cells and prevention of pathogenesis. Thus, to determine the effect of ALA-PDT on T cell-based immunity, these paired cases were studied by co-staining with CD3 and CD8. We found an increase in the number of CD3^+^ T cells and CD8^+^ T cell infiltration in CIN2 compared to that before ALA-PDT (**Figures 1A, B**). Moreover, at the 6-month follow-up, the number of CD8^+^ T cell subsets was significantly higher in the HPV clearance group than in the HPV-positive group (**Figure 1C**). The number of CD8^+^ T cells was also significantly higher in the CIN2 disappearance group than that in the regression group (**Figure 1D**).

CD8^+^ T cells attack HPV-infected cells by releasing GrB, a marker used to identify activated cytotoxic T cells (13). Therefore, we evaluated the expression of GrB in cytotoxic CD8^+^ T cells to understand their activation states and found an increase in the percentage of CD8^+^ T cells expressing GrB in post-treatment samples (**Figures 1E, F**). Furthermore, the proportion of CD8^+^ T cells expressing GrB was significantly higher in the HPV clearance group than that in the HPV-positive group (**Figure 1G**). In addition, the CIN2 disappearance group showed higher GrB expression in CD8^+^ T cells than the regression group (**Figure 1H**). These data indicate that ALA-PDT can activate CD8^+^ T-cell immunity to eliminate abnormal cervical cells.

### 3.3 ALA-PDT increases CD8^+^ T cell infiltration by regulating CXCR3

**Figure 2:**
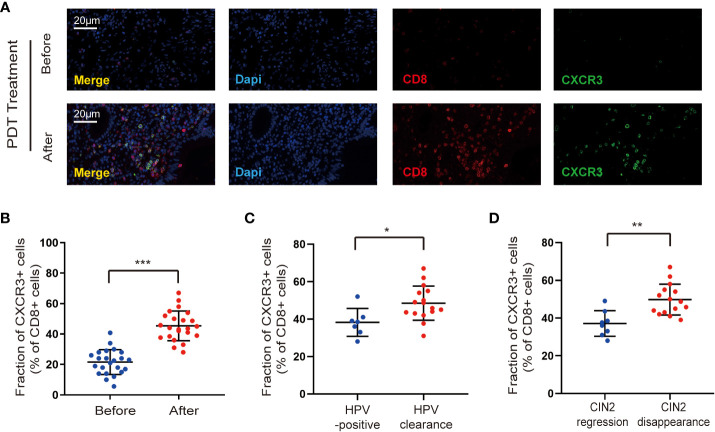
ALA-PDT treatment increases CD8^+^ T cell infiltration by regulating CXCR3. **(A, B)** There is an increase in CXCR3 expression on CD8^+^ T cells after ALA-PDT treatment compared to that before treatment. **(C)** At the end of the 6-month follow-up, the proportion of CD8^+^ T cells expressing CXCR3 was significantly higher in the HPV clearance group than in the HPV-positive group. **(D)** The CIN2 disappearance group exhibited increased CXCR3 expression by CD8^+^ T cells than the CIN2 regression group. Differences between linked groups were evaluated by two-tailed Student’s t test. ^*^p<0.05; ^**^p<0.01; ^***^p < 0.001.

Understanding why ALA-PDT promotes CD8^+^ T cell entry into lesions is an important advancement in efforts to harness the ability of the immune system to fight CIN2. CXCR3 is critical for the trafficking and activation of CD8^+^ T cells (14), thus we detected the change in CXCR3 expression on CD8+ T cells after ALA-PDT. Our data showed a remarkable increase in CXCR3 expression on CD8^+^ T cells after ALA-PDT compared to that before treatment in patients with CIN2 (**Figures 2A, B**). Furthermore, at the end of the 6-month follow-up period, the proportion of CD8^+^ T cells expressing CXCR3 was significantly higher in the HPV clearance group than in the HPV-positive group (**Figure 2C**). In addition, the CIN2 disappearance group exhibited increased CXCR3 expression in CD8^+^ T cells compared to the regression group (**Figure 2D**). These data suggest that ALA-PDT increases CD8^+^ T cell infiltration by promoting CXCR3 expression.

### 3.4 The effect of ALA-PDT on CD8^+^ memory T cells

Memory CD8^+^ T cells are capable of persisting and functioning throughout cervical lesions and can rapidly differentiate into effector CD8^+^ T cells upon pathogen re-encounter to eliminate HPV infection. Our data showed a significant increase in the infiltration of CD8^+^ T cells after ALA-PDT; therefore, we investigated whether ALA-PDT promotes the infiltration of CD8^+^ T cells by regulating the CD8^+^ memory T cell subset.

**Figure 3:**
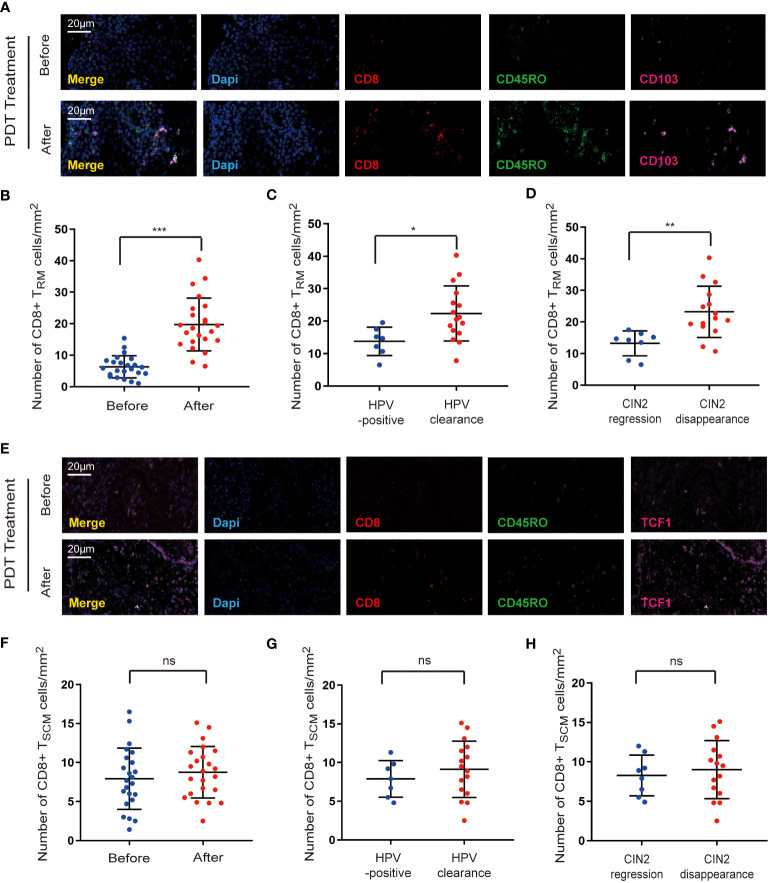
The effect of ALA-PDT treatment on CD8^+^ memory T cells. **(A, B)** Cervical lesion showed a higher number of CD8^+^ T_RM_ cells (CD8^+^CD45RO^+^CD103^+^) after ALA-PDT treatment than before ALA-PDT treatment. **(C)** The density of CD8^+^ T_RM_ cells was higher in the HPV clearance group than in the HPV-positive group. **(D)** The density of CD8^+^ T_RM_ cells was higher in the CIN2 disappearance group compared to the CIN2 regression group. **(E, F)** The amount of CD8^+^ T_SCM_ cells (CD8^+^CD45RO^+^TCF1^+^) do not show obvious changes after ALA-PDT treatment. **(G)** There is no significant difference in the number of CD8^+^ T_SCM_ cells between the HPV clearance group and the HPV-positive group. **(H)** No significant difference was found in the number of CD8^+^ T_SCM_ cells between the CIN2 disappearance and regression groups. Differences between linked groups were evaluated by two-tailed Student’s t test. ^*^p<0.05; ^**^p<0.01; ^***^p < 0.001; ns not significant.

Tissue-resident memory T (T_RM_) cells were identified as a subset of memory T cells (15, 16), and we first detected the infiltration of CD8^+^ T_RM_ cells into the paired samples by co-staining for CD8, CD45RO, and CD103. As shown in **Figures 3A, B**, cervical lesions showed a higher number of CD8^+^ T_RM_ cells after ALA-PDT. Furthermore, the density of CD8^+^ T_RM_ cells was higher in the HPV clearance group than that in the HPV-positive group (**Figure 3C**). In addition, an increase in CD8^+^ T_RM_ cell infiltration was observed in the CIN2 disappearance group compared to that in the regression group (**Figure 3D**).

Stem-like cell memory T (T_SCM_) cells constitute another subset of memory T cells (17), and we further examined the distribution of CD8^+^ T_SCM_ cells by co-staining for CD8, CD45RO, and TCF1. Our data revealed that the number of CD8^+^ T_SCM_ cells did not show any obvious change after ALA-PDT (**Figures 3E, F**). Moreover, there was no significant difference in the number of CD8^+^ T_SCM_ cells between the HPV clearance and HPV-positive groups (**Figure 3G**). In addition, no significant difference was observed in the number of CD8^+^ T_SCM_ cells between the CIN2 disappearance and regression groups (**Figure 3H**).

### 3.5 ALA-PDT down-regulates PD-1 expression on CD8^+^ T cells

**Figure 4:**
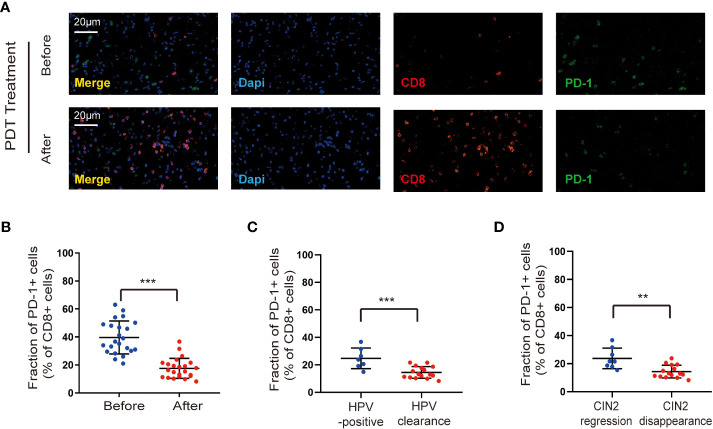
ALA-PDT treatment down-regulates PD-1 expression on CD8^+^ T cells. **(A, B)** Cervical lesions with a decreased percentage of CD8^+^ T cells expressing PD-1 were detected after ALA-PDT treatment compared to before treatment. **(C)** The HPV clearance group showed a reduction in the proportion of CD8^+^ T cells expressing PD-1 compared to the HPV-positive group after ALA-PDT treatment. **(D)** The CIN2 disappearance group showed a decreased percentage of CD8^+^ T cells expressing PD-1 compared to the CIN2 regression group. Differences between linked groups were evaluated by two-tailed Student’s t test. ^**^p<0.01; ^***^p < 0.001.

PD-1 has been identified as a negative regulator of T cell activity (18). To better understand the mechanism by which ALA-PDT promotes CD8^+^ T immunity, we determined the proportion of CD8^+^ T cells expressing PD-1. After ALA-PDT, cervical lesions with a decreased percentage of CD8^+^ T cells expressing PD-1 were detected compared with those before treatment (**Figures 4A, B**). Moreover, the HPV clearance group showed a reduction in the proportion of CD8^+^ T cells expressing PD-1 compared to that in the HPV-positive group after ALA-PDT (**Figure 4C**). In addition, the CIN2 disappearance group showed a decreased percentage of CD8^+^ T cells expressing PD-1 compared with the regression group (**Figure 4D**).

## 4 Discussion

ALA-PDT is a modern and non-invasive form of therapy applied in the treatment of non-oncological diseases, as well as cancers of various types and locations. It has been verified that ALA-PDT activates multiple mechanisms of cell death by generating oxidative stress. Most prior studies have shown that ALA-PDT elicits antitumor immune responses with systemic or local effects (19, 20). Recently, it was reported that the number of CD8^+^ T cells increased after ALA-PDT in patients with CIN2 (21, 22). Consistent with these studies, we observed that the local immune response was activated after ALA-PDT in patients with CIN2, presenting increased CD45^+^, CD3^+^, and CD8^+^ T cell infiltration. In addition, our study revealed that ALA- PDT enhances the cytotoxicity of CD8^+^ T cells by promoting GrB expression. We first evaluated the relationship between the local immune response and clinical efficacy of ALA-PDT in patients with CIN2. Notably, the number of CD8^+^ T cell subsets was significantly higher in the HPV clearance group than in the HPV-positive group, and the infiltration of CD8^+^ T cells was higher in the CIN2 disappearance group than in the CIN2 regression group. In addition, the expression of GrB in CD8^+^T cells was higher in the HPV clearance and CIN2 disappearance groups than in the HPV-positive and CIN2 regression groups. These results suggest that ALA-PDT enhances CD8^+^ T-cell responses, which protect the host against CIN2 (23).

CXCR3 is primarily expressed on CD8^+^ T cells and is of paramount importance for the recruitment of CD8^+^ T cells. And increased expression of CXCR3 in CD8^+^T cells promotes the trafficking of CD8^+^ T cells to lesions (24). Similarly, our data showed that the expression of CXCR3 in CD8^+^ T cells was increased in patients with CIN2 after ALA-PDT. Furthermore, higher CXCR3 expression of CD8^+^ T cells was detected in the HPV clearance and CIN2 disappearance groups than in the HPV-positive and CIN2 regression groups. These results revealed that ALA-PDT may promote CD8^+^ T cell responses by upregulating CXCR3 expression. In addition, CXCR3 is a chemokine receptor with three ligands, CXCL9, CXCL10, and CXCL11 (25). Whether ALA-PDT could regulate the expression of these chemokines is still unknown in patients with CIN2; however, we did not examine chemokine expression after ALA-PDT in this study (26).

Emerging data suggest that memory CD8^+^ T cells constitute the immunological memory to provide long-term protection (27) Memory CD8^+^ T cells, especially a subclass of CD8^+^ T_RM_ cells that mainly reside in barrier tissues at interfaces, are crucial effector cells in local immune responses that rapidly proliferate and provide a long-term localized defense against pathogens. To our knowledge, this is the first study to explore the effects of ALA-PDT on CD8^+^ T_RM_ cells. Our data revealed that ALA-PDT promoted CD8^+^ T_RM_ cell infiltration. Patients with higher infiltration of CD8^+^ T_RM_ cells were able to eliminate HPV infection and CIN2 more easily. CD8^+^ T_SCM_ cells, another subset of memory CD8^+^T cells with the ability to self-renew, can generate short-lived effector memory T cells that are involved in killing target cells. Stem-like cell memory T (T_SCM_) cells constitute another subset of memory T cells (28). Therefore, we investigated the expression of CD8^+^ T_SCM_ cells after ALA-PDT and found that the infiltration of CD8^+^ T_SCM_ cells did not change significantly after ALA-PDT. This result may be due to the small sample size or changes in CD8^+^ T_SCM_ cells occurring in a short time. At the 6-month follow-up, CD8^+^ T_SCM_ cells were no longer the main functional subpopulation. A larger sample size and shorter follow-up period should be considered in future studies. Overall, ALA-PDT induces an increase in the number of CD8^+^ T_RM_ cells, contributing to long-term therapeutic effects.

The host immune response to viruses is mediated by inhibitory ligands and receptors on immune cells. PD-1, mainly expressed on activated T cells, plays a crucial role in inhibiting immune responses and promoting self-tolerance by modulating T cell activity and activating apoptosis in antigen-specific T cells (29). Moreover, PD-1 overexpression correlates with T cell exhaustion (30). Presently, most studies have focused on the combination of PDT and PD-1 checkpoint blockade to induce potent antitumor efficacy (31, 32). We were curious about the influence of ALA-PDT on PD-1 expression in CD8^+^ T cells in CIN2, as there been no reports on this. In our study, we examined the effect of ALA-PDT on the expression of PD-1 in CD8^+^ T cells and found that PD-1 expression in CD8^+^ T cells was downregulated after ALA-PDT. Patients with HPV-positive or CIN2 regression showed higher PD-1 expression in CD8^+^ T cells than those with HPV clearance or CIN2 disappearance. These findings indicate that ALA-PDT enhances the CD8+ T cell response by regulating the expression of PD-1 in CD8^+^ T cells.

Collectively, our findings successfully demonstrated that ALA-PDT could activate CD8^+^ T cell responses by modulating the expression of CXCR3 and PD-1 in CD8^+^ T cells and increasing the infiltration of CD8^+^ T_RM_ cells. In addition, the infiltration of CD8^+^ T cells is correlated with the prognosis of CIN2.

## Data availability statement

The original contributions presented in the study are included in the article/supplementary material. Further inquiries can be directed to the corresponding authors.

## Ethics statement

The studies involving humans were approved by Ethics Committee of Renji Hospital of Shanghai Jiaotong University. The studies were conducted in accordance with the local legislation and institutional requirements. The participants provided their written informed consent to participate in this study.

## Author contributions

AW and JN performed the study. ZH and LG enrolled the patients. AW, JN and LQ analyzed the data, and prepared the manuscript. LQ designed and supervised the study. All authors contributed to the article and approved it for publication.
